# Supplementary material for: Third primary SARS-CoV-2 mRNA vaccines enhance antibody responses in most patients with haematological malignancies
Source: Nat Commun. 2022 Nov 14;13:6922. doi: 10.1038/s41467-022-34657-z (PMC9662771; doi:10.1038/s41467-022-34657-z)
Supplement: Supplementary file 1 — Supplementary Information [file 41467_2022_34657_MOESM1_ESM.pdf]

Third primary SARS-CoV-2 mRNA vaccines enhance antibody responses in most patients with haematological malignancies

Supplemental data

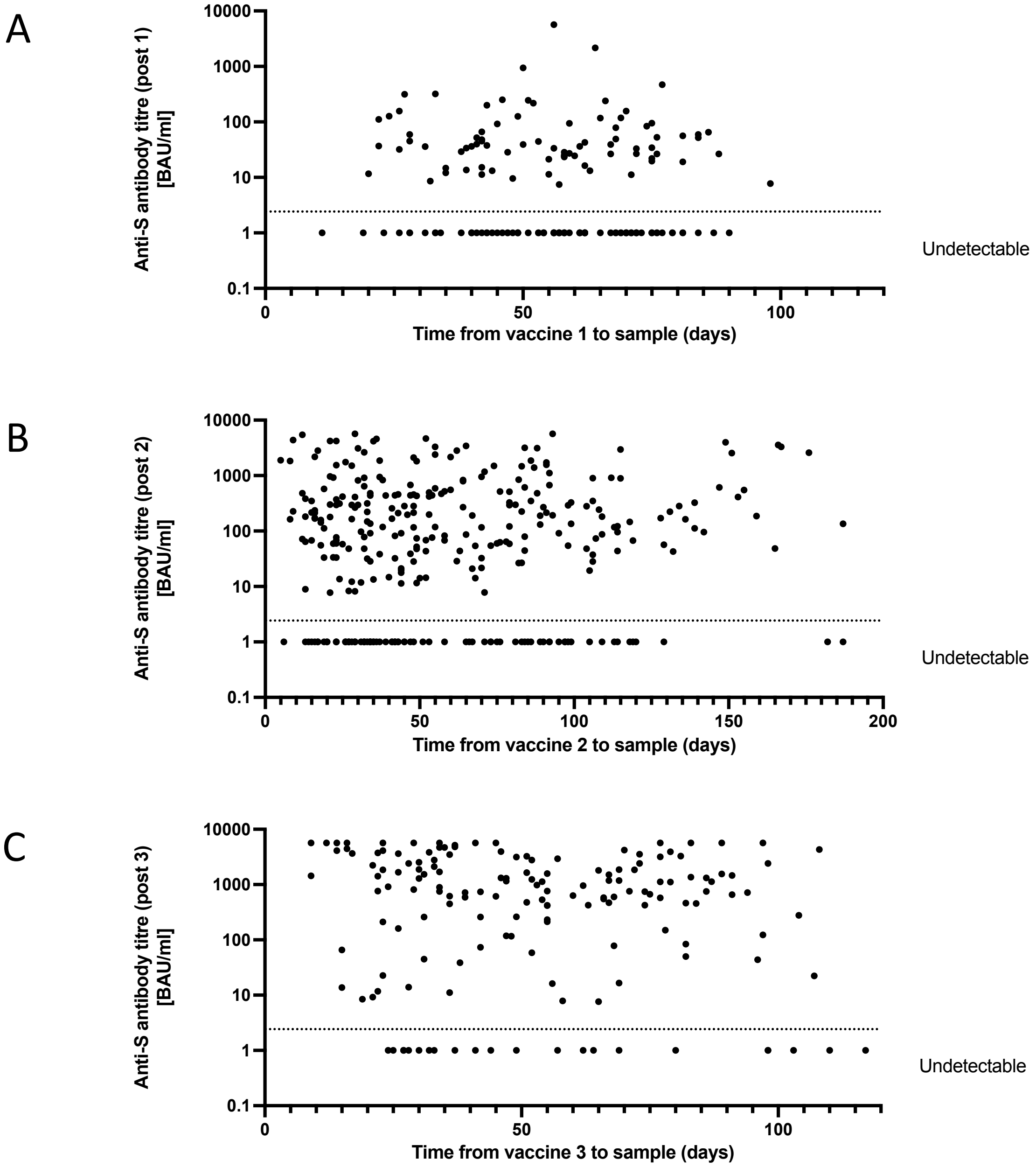

Supplemental figure 1. Antibody sampling timepoints. **a** Scatter plot graphs showing anti-S antibody titers vs time of sample collection post vaccine 1 , **b** post vaccine 2, and **c** post vaccine 3, in patients with haematological malignancies. Note that undetectable values (<7.1 BAU/mL) are shown as 1 for illustrative purposes only. BAU, binding antibody units.

Supplemental data

A

	% Detectable Post 1	% Detectable Post 2	% Detectable Post 3	% >568 Post 1	% >568 Post 2	% >568 Post 3
**Acute Leukaemia/MDS**	50.0	90.0	100.0	0.0	10.0	60.0
**Allograft <1y**	45.5	54.5	100.0	0.0	36.4	80.0
**Allograft >1y**	61.8	85.9	96.6	0.0	29.7	86.2
**CLL**	5.6	24.4	52.6	0.0	4.9	21.1
**Lymphoma (B cell)**	36.4	53.3	60.9	4.5	8.9	21.7
**Lymphoma (Hodgkin)**	66.7	66.7	100.0	11.1	33.3	75.0
**Lymphoma (PTLD)**	25.0	66.7	100.0	0.0	0.0	66.7
**MPN (ET)**	79.2	98.0	100.0	0.0	22.0	81.2
**MPN (Myelofibrosis)**	37.5	67.9	92.9	0.0	7.1	57.1
**MPN (PV)**	45.5	91.7	94.7	0.0	27.8	63.2
**Plasma Cell Dyscrasia**	46.7	95.0	85.7	6.7	25.0	28.6
**Other**	20.0	70.0	100.0	0.0	20.0	50.0
**Overall**	47.4	73.1	86.4	1.8	19.3	58.6
**Healthy Controls**	100.0	100.0		3.6	55.3	

Colour scale: 0, 20, 40, 60, 80, 100

B

	% Detectable Post 1 (95% CI) [Raw data]	% Detectable Post 2 (95% CI) [Raw data]	% Detectable Post 3 (95% CI) [Raw data]	% > 568 Post 1 (95% CI) [Raw data]	% > 568 Post 2 (95% CI) [Raw data]	% > 568 Post 3 (95% CI) [Raw data]
Acute Leukaemia/MDS	50.0 (1.3-98.7) [N=1/2]	90.0 (55.5-99.8) [N=9/10]	100.0 (47.8-100.0) [N=5/5]	0.0 (0.0-84.2) [N=0/2]	10.0 (0.0-44.5) [N=1/10]	60.0 (14.7-94.7) [N=3/5]
Allograft <1y	45.4 (16.8-76.6) [N=5/11]	54.5 (23.4-83.2) [N=6/11]	100.0 (47.8-100.0) [N=5/5]	0.0 (0.0-28.5) [N=0/11]	36.4 (10.9-69.2) [N=4/11]	80.0 (28.4-99.5) [N=4/5]
Allograft >1y	61.8 (43.6-77.8) [N=21/34]	85.9 (75.0-93.4) [N=55/64]	96.6 (82.2-99.9) [N=28/29]	0.0 (0.0-10.3) [N=0/34]	29.7 (18.9-42.4) [N=19/64]	86.2 (68.3-96.1) [N=25/29]
CLL	5.6 (0.1-27.3) [N=1/18]	24.4 (12.4-40.3) [N=10/41]	52.6 (28.9-75.6) [N=10/19]	0.0 (0.0-18.5) [N=0/18]	4.9 (0.6-16.5) [N=2/41]	21.1 (6.0-45.6) [N=4/19]
Lymphoma (B cell)	36.4 (17.2-59.3) [N=8/22]	53.3 (37.9-68.3) [N=24/45]	60.9 (38.5-80.3) [N=14/23]	4.5 (9.1-22.8) [N=1/22]	8.9 (2.5-21.2) [N=4/45]	21.7 (7.5-43.7) [N=5/23]
Lymphoma (Hodgkin)	66.7 (29.9-92.5) [N=6/9]	66.7 (29.9-92.5) [N=6/9]	100.0 (39.8-100.0) [N=4/4]	11.1 (0.2-48.3) [N=1/9]	33.3 (7.5-70.1) [N=3/9]	75.0 (19.4-99.4) [N=3/4]
Lymphoma (PTLD)	25.0 (0.6-80.6) [N=1/4]	66.7 (9.4-99.2) [N=2/3]	100.0 (29.2-100.0) [N=3/3]	0.0 (0.0-60.2) [N=0/4]	0.0 (0.0-70.8) [N=0/3]	66.7 (9.4-99.2) [N=2/3]
MPN (ET)	79.2 (57.8-92.9) [N=19/24]	98.0 (89.4-100.0) [N=49/50]	100.0 (89.1-100.0) [N=32/32]	0.0 (0.0-14.3) [N=0/24]	22.0 (11.5-36) [N=11/50]	81.2 (63.6-92.8) [N=26/32]
MPN (Myelofibrosis)	37.5 (15.2-64.6) [N=6/16]	67.9 (47.6-84.1) [N=19/28]	92.9 (66.1-99.8) [N=13/14]	0.0 (0.0-20.6) [N=0/16]	7.1 (0.8-23.5) [N=2/28]	57.1 (28.9-82.3) [N=8/14]
MPN (PV)	45.5 (16.8-76.6) [N=5/11]	91.7 (77.5-98.2) [N=33/36]	94.7 (74.0-99.9) [N=18/19]	0.0 (0.0-28.5) [N=0/11]	27.8 (14.2-45.2) [N=10/36]	63.2 (38.4-83.7) [N=12/19]
Plasma Cell Dyscrasia	46.7 (21.3-73.4) [N=7/15]	95.0 (75.1-99.9) [N=19/20]	85.7 (42.1-99.6) [N=6/7]	6.7 (0.2-32) [N=1/15]	25.0 (0.9-49.1) [N=5/20]	28.6 (3.7-71.0) [N=2/7]
Other	20.0 (0.5-71.6) [N=1/5]	70.0 (34.8-93.3) [N=7/10]	100.0 (15.8-100.0) [N=2/2]	0.0 (0.0-52.2) [N=0/5]	20.0 (2.5-55.6) [N=2/10]	50.0 (1.3-98.7) [N=1/2]
Overall	47.4 (39.7-55.1) [N=81/171]	73.1 (67.9-77.8) [N=239/327]	86.4 (80.2-91.3) [N=140/162]	1.8 (0.4-5.0) [N=3/171]	19.3 (15.1-24.0) [N=63/327]	58.6 (50.6-66.3) [N=95/162]
Healthy Controls	100.0 (87.7-100.0) [N=28/28]	100.0 (92.4-100.0) [N=47/47]	NA	3.6 (0.0-18.4) [N=1/28]	55.3 (40.1-69.8) [N=26/47]	NA

Supplemental figure 2. Effect of disease on serological response. a Heatmap showing serological responses to SARS-CoV-2 vaccination in patients with haematological malignancies stratified by disease, values represent percentage of patient with detectable anti-S antibodies (>7.1 BAU/ml), and antibody responses greater than the bottom 10% of the assay range (>568 BAU/ml). **b** Table of percentages (with 95% confidence intervals (CI)), and [raw data]. MDS, myelodysplastic syndrome, CLL, chronic lymphocytic leukaemia, PTLD, post-transplant lymphoproliferative disorder, MPN, myeloproliferative neoplasm, ET, essential thrombocythemia, PV, polycythaemia vera.

Supplemental data

A

	% Detectable Post 1	% Detectable Post 2	% Detectable Post 3	% >568 Post 1	% >568 Post 2	% >568 Post 3
BTKi	0.0	15.4	37.5	0.0	0.0	0.0
Chemo (<6m)	33.3	80.0	90.9	4.8	16.7	36.4
Hydroxycarbemide	57.9	94.3	97.0	0.0	20.8	69.7
Interferon	85.7	100.0	100.0	0.0	33.3	60.0
JAKi	18.2	57.1	90.9	0.0	4.8	36.4
None	66.7	83.1	96.3	0.0	22.0	81.5
PTx-Chemo	33.3	66.7	100.0	0.0	33.3	0.0
PTx-Immunosuppression	36.8	63.0	94.1	0.0	22.2	76.5
PTx-Off Immunosuppression	82.4	96.9	100.0	0.0	40.6	100.0
PTx-TKI	80.0	90.9	100.0	0.0	18.2	100.0
R/O-Chemo (<6m)	27.8	40.5	55.0	5.6	8.1	15.0
R/O-Chemo (6-24m)	40.0	60.0	100.0	20.0	20.0	100.0
R/O-Chemo (>24m)	50.0	90.9	100.0	0.0	9.1	60.0
Venetoclax	0.0	11.1	50.0	0.0	0.0	25.0
Other	57.1	57.1	50.0	0.0	42.9	50.0
Overall	47.4	73.1	86.4	1.8	19.3	58.6
Healthy Controls	100.0	100.0		3.6	55.3	

Colour scale: 0 – 20 – 40 – 60 – 80 – 100

B

	% Detectable Post 1 (95% CI) [Raw data]	% Detectable Post 2 (95% CI) [Raw data]	% Detectable Post 3 (95% CI) [Raw data]	% > 568 Post 1 (95% CI) [Raw data]	% > 568 Post 2 (95% CI) [Raw data]	% > 568 Post 3 (95% CI) [Raw data]
BTKi	0.0 (0.0-30.8) [N=0/10]	15.4 (1.9-45.4) [N=2/13]	37.5 (8.5-75.5) [N=3/8]	0.0 (0.0-30.8) [N=0/10]	0.0 (0.0-24.7) [N=0/13]	0.0 (0.0-36.9) [N=0/8]
Chemo (<6m)	33.3 (14.5-57.0) [N=7/21]	80.0 (61.4-92.3) [N=24/30]	90.9 (58.7-99.8) [N=10/11]	4.8 (0.1-23.8) [N=1/21]	16.7 (5.6-34.7) [N=5/30]	36.4 (10.9-69.2) [N=4/11]
Hydroxycarbemide	57.9 (33.5-79.8) [N=11/19]	94.3 (84.3-98.8) [N=50/53]	97.0 (84.2-99.9) [N=32/33]	0.0 (0.0-17.6) [N=0/19]	20.8 (10.8-34.1) [N=11/53]	69.7 (51.3-84.4) [N=23/33]
Interferon	85.7 (42.1-99.6) [N=6/7]	100.0 (66.4-100.0) [N=9/9]	100.0 (47.8-100.0) [N=5/5]	0.0 (0.0-41.0) [N=0/7]	33.3 (7.5-70.1) [N=3/9]	60.0 (14.7-94.7) [N=3/5]
JAKi	18.2 (2.3-51.8) [N=2/11]	57.1 (34.0-78.2) [N=12/21]	90.9 (58.7-99.8) [N=10/11]	0.0 (0.0-28.5) [N=0/11]	4.8 (0.1-23.8) [N=1/21]	36.4 (10.9-69.2) [N=4/11]
None	66.7 (44.7-84.4) [N=16/24]	83.1 (71.0-91.6) [N=49/59]	96.3 (81.0-99.9) [N=26/27]	0.0 (0.0-14.2) [N=0/24]	22.0 (12.3-34.7) [N=13/59]	81.5 (61.9-93.7) [N=22/27]
PTx-Chemo	33.3 (0.0-90.6) [N=1/3]	66.7 (9.4-99.2) [N=2/3]	100.0 (2.5-100.0) [N=1/1]	0.0 (0.0-70.8) [N=0/3]	33.3 (0.8-90.6) [N=1/3]	0.0 (0.0-97.5) [N=0/1]
PTx-Immunosuppression	36.8 (16.3-61.6) [N=7/19]	63.0 (42.4-80.6) [N=17/27]	94.1 (71.3-99.8) [N=16/17]	0.0 (0.0-17.6) [N=0/19]	22.2 (8.8-42.3) [N=6/27]	76.5 (50.1-93.2) [N=13/17]
PTx-Off Immunosuppression	82.4 (56.6-96.2) [N=14/17]	96.9 (83.8-99.9) [N=31/32]	100.0 (69.1-100.0) [N=10/10]	0.0 (0.0-19.5) [N=0/17]	40.6 (23.7-59.4) [N=13/32]	100.0 (69.2-100.0) [N=10/10]
PTx-TKI	80.0 (28.4-99.5) [N=4/5]	90.9 (58.7-99.8) [N=10/11]	100.0 (54.1-100.0) [N=6/6]	0.0 (0.0-52.2) [N=0/5]	18.2 (2.3-51.8) [N=2/11]	100.0 (54.1-100.0) [N=6/6]
R/O-Chemo (<6m)	27.8 (9.7-53.5) [N=5/18]	40.5 (24.8-57.9) [N=15/37]	55.0 (31.5-76.9) [N=11/20]	5.6 (0.1-27.3) [N=1/18]	8.1 (1.7-21.9) [N=3/37]	15.0 (3.2-37.9) [N=3/20]
R/O-Chemo (6-24m)	40.0 (5.3-85.4) [N=2/5]	60.0 (14.7-94.7) [N=3/5]	100.0 (15.8-100.0) [N=2/2]	20.0 (0.5-71.6) [N=1/5]	20.0 (0.5-71.6) [N=1/5]	100.0 (15.8-100.0) [N=2/2]
R/O-Chemo (>24m)	50.0 (6.7-93.2) [N=2/2]	90.9 (58.7-99.8) [N=10/11]	100.0 (47.8-100.0) [N=5/5]	0.0 (0.0-60.2) [N=0/4]	9.1 (0.2-41.3) [N=1/11]	60.0 (14.7-94.7) [N=3/5]
Venetoclax	0.0 (0.0-97.5) [N=0/1]	11.1 (0.2-48.2) [N=1/9]	50.0 (6.7-93.2) [N=2/4]	0.0 (0.0-97.5) [N=0/1]	0.0 (0.0-33.6) [N=0/9]	25.0 (0.6-80.6) [N=1/4]
Other	57.1 (18.4-90.1) [N=4/7]	57.1 (18.4-90.1) [N=4/7]	50.0 (1.3-98.7) [N=1/2]	0.0 (0.0-41.0) [N=0/7]	42.9 (9.9-81.6) [N=3/7]	50.0 (1.3-98.7) [N=1/2]
Overall	47.4 (39.7-55.1) [N=81/171]	73.1 (67.9-77.8) [N=239/327]	86.4 (80.2-91.3) [N=140/162]	1.8 (0.3-5.0) [N=3/171]	19.3 (15.1-24.0) [N=63/327]	58.6 (50.6-66.3) [N=95/162]
Healthy Controls	100.0 (87.7-100.0) [N=28/28]	100.0 (92.4-100.0) [N=47/47]	NA	3.6 (0.0-18.4) [N=1/28]	55.3 (40.1-69.8) [N=26/47]	NA

Supplemental figure 3. Effect of treatment on serological response. a Heatmap showing serological responses to SARS-CoV-2 vaccination in patients with haematological malignancies stratified by treatment, values represent percentage of patient with detectable anti-S antibodies (>7.1 BAU/ml), and antibody responses greater than the bottom 10% of the assay range (>568 BAU/ml). **b** Table of percentages (with 95% confidence intervals (CI)) and [raw data] . BTKi, burton tyrosine kinase inhibitor, Chemo, cytotoxic chemotherapy, JAKi, Jak-stat inhibitor, PTx, post allogeneic stem cell transplant, TKI, tyrosine kinase inhibitor, R/O, rituximab or obinotuzumab.

Supplemental data

A

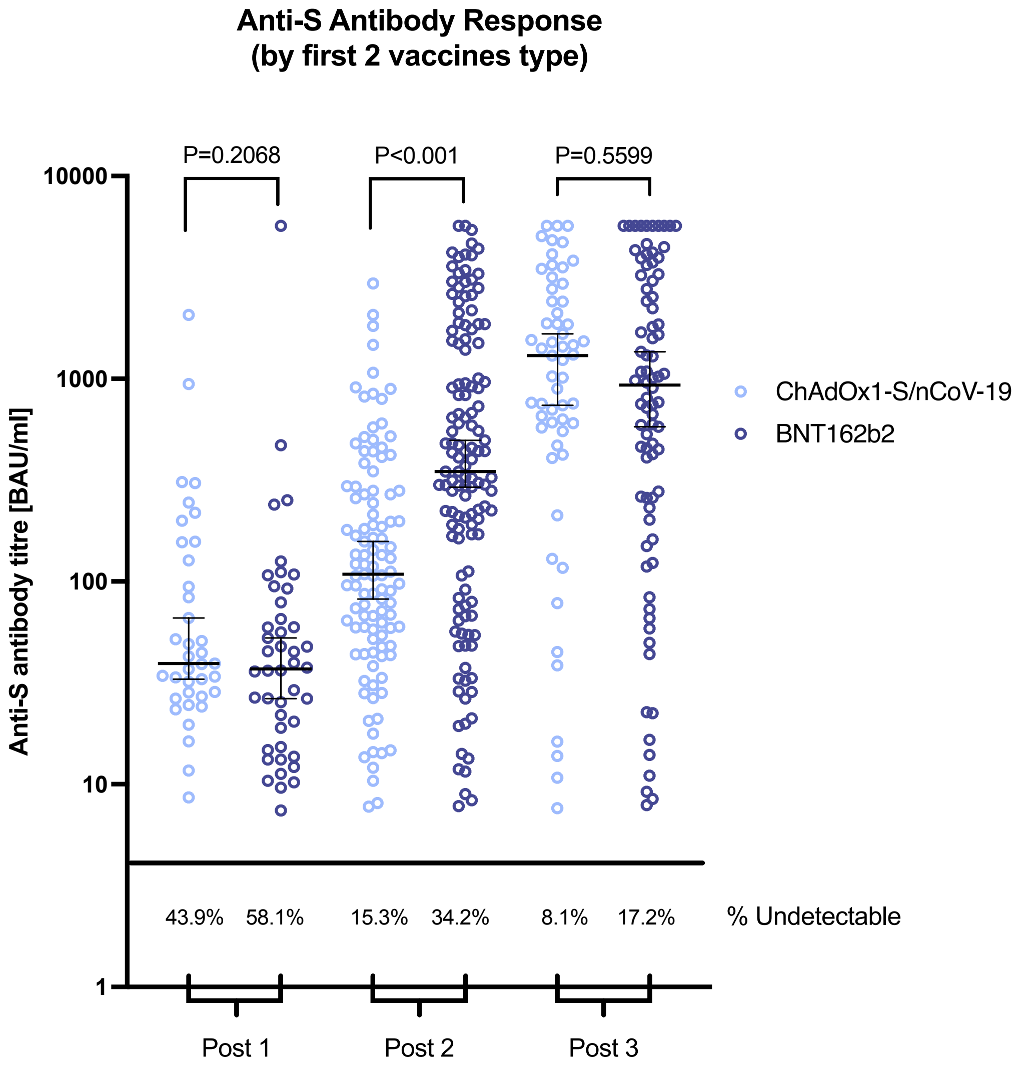

Supplemental figure 4. Effect of vaccine type on serological response. a Dot plot of anti-S antibody titers post 1 (ChAdOx1-S, N=66, BNT162b2 N=105), 2 (ChAdOx1-S, N=124, BNT162b2 N=199), and 3 vaccines (ChAdOx1-S, N=62, BNT162b2 N=99). Bars represent median and 95% confidence intervals. Groups compared by Mann-Whitney U test. BAU, binding antibody units.
